# Supplementary material for: The equatorial position of the metaphase plate ensures symmetric cell divisions
Source: eLife. 2015 Jul 18;4:e05124. doi: 10.7554/eLife.05124 (PMC4536468; doi:10.7554/eLife.05124)
Supplement: Source code 1. — Custom built software in Matlab. DOI: http://dx.doi.org/10.7554/eLife.05124.021 [file elife05124s001.zip › Poles and Kinetochores/External/geom3d/geom3d-demos/html/demoDrawTubularMesh.html]

demoDrawTubularMesh 

## Contents

- Initialisation
- Draw each edge as a green cylinder
- Draw each vertex as a blue ball
- More setup on display

```
function demoDrawTubularMesh(varargin)
```

```
%DEMODRAWTUBULARMESH  One-line description here, please.
%
%   output = demoDrawTubularMesh(input)
%
%   Example
%   demoDrawTubularMesh
%
%   See also
%
%
% ------
% Author: David Legland
% e-mail: david.legland@grignon.inra.fr
% Created: 2012-04-25,    using Matlab 7.9.0.529 (R2009b)
% Copyright 2012 INRA - Cepia Software Platform.
```

## Initialisation

```
% extract vertices, edges, and faces of soccerball polyhedron
[vertices edges faces] = createSoccerBall;

% prepare figure
figure(1); clf; hold on;
axis equal;
view(3);

% draw the polyhedron as basis
drawPolyhedron(vertices, faces);
```

## Draw each edge as a green cylinder

```
figure; hold on;
axis equal;
view(3);

width = .05;
radius = .1;

for i = 1:size(edges, 1)
    p1 = vertices(edges(i, 1), :);
    p2 = vertices(edges(i, 2), :);

    drawCylinder([p1 p2 width], 'FaceColor', 'g');
end
```

## Draw each vertex as a blue ball

```
for i = 1:size(vertices, 1)
    drawSphere([vertices(i, :) radius], 'FaceColor', 'b');
end
```

## More setup on display

```
set(gcf, 'renderer', 'opengl')
light;
view([10 30]);
```

Published with MATLAB® 7.9
