# Supplementary material for: The equatorial position of the metaphase plate ensures symmetric cell divisions
Source: eLife. 2015 Jul 18;4:e05124. doi: 10.7554/eLife.05124 (PMC4536468; doi:10.7554/eLife.05124)
Supplement: Source code 1. — Custom built software in Matlab. DOI: http://dx.doi.org/10.7554/eLife.05124.021 [file elife05124s001.zip › Poles and Kinetochores/External/geom3d/geom3d-demos/html/demoGeom3d.html]

demoGeom3d 

## Contents

- Create points and lines
- Create planes
- Compute intersections

```
function demoGeom3d(varargin)
```

```
%DEMOGEOM3D Demo of geom3d basic processing
%
%   Usage:
%   demoGeom3d
%
%   This demo demonstrates some usage example of the geom3d library.
%   In particular, it shows how to:
%   - create shapes like points, edges, lines and plane
%   - draw these shape on current figure
%   - compute intersections of lines and planes
%   - compute lines perpendicular to a plane
%
%   See also
%
%
% ------
% Author: David Legland
% e-mail: david.legland@grignon.inra.fr
% Created: 2009-06-22,    using Matlab 7.7.0.471 (R2008b)
% Copyright 2009 INRA - Cepia Software Platform.
```

## Create points and lines

```
% create some points: points are simply represented by a row vector of 3
% elements.
p1 = [10 20 30];
p2 = [80 10 20];
p3 = [20 50 10];

% points can be grouped into arrays to represent point sets
points = [p1;p2;p3];

% create a 3D line through 2 points
line12 = createLine3d(p1, p2);

% an edge is represented by concatenating its vertices
edge13 = [p1 p3];

% prepare a figure for drawing
figure(1); clf; hold on;
axis equal;
axis([0 100 0 100 0 100]);
set(gcf, 'renderer', 'opengl');
set(gca, 'CameraPosition', [400 -200 800]);

% draw all points
drawPoint3d(points);

% draw the line, with some drawing options (clipping is automatic).
drawLine3d(line12, 'color', 'k');

% draw an edge, using a thick stroke and a different color
drawEdge(edge13, 'color', 'r', 'linewidth', 2);
```

## Create planes

```
% create a plane from 3 points
plane = createPlane(p1, p2, p3);

% draw the plane. Clipping is automatically performed.
drawPlane3d(plane);

% create a line from a point and a direction vector
p0 = [60 60 60];
n0 = [-10 1 10];
plane0 = createPlane(p0, n0);

% draw the plane, changing its color
drawPlane3d(plane0, 'g');
```

## Compute intersections

```
% compute intersection between 2 planes
line = intersectPlanes(plane0, plane);
drawLine3d(line, 'lineWidth', 2);

% compute intersection between a plane and a line
inter = intersectLinePlane(line12, plane0);
drawPoint3d(inter, 'marker', '+', 'markerSize', 10, 'linewidth', 3);

% create a line perpendicular to a plane
normal = planeNormal(plane);
perpLine = [80 60 40 normal];
drawLine3d(perpLine);

% compute intersection of line with plane
inter2 = intersectLinePlane(perpLine, plane);
drawPoint3d(inter2);
```

Published with MATLAB® 7.9
