# Supplementary material for: The equatorial position of the metaphase plate ensures symmetric cell divisions
Source: eLife. 2015 Jul 18;4:e05124. doi: 10.7554/eLife.05124 (PMC4536468; doi:10.7554/eLife.05124)
Supplement: Source code 1. — Custom built software in Matlab. DOI: http://dx.doi.org/10.7554/eLife.05124.021 [file elife05124s001.zip › Poles and Kinetochores/External/geom3d/geom3d-demos/html/demoInertiaEllipsoid.html]

demoInertiaEllipsoid 

## Contents

- Data generation
- Inertia ellipsoid computation and display

```
function demoInertiaEllipsoid(varargin)
```

```
%DEMOINERTIAELLIPSOID Demo program for the use of ellipsoids
%
%   Usage:
%   demoInertiaEllipsoid;
%
%   Example
%   demoInertiaEllipsoid
%
%   See also
%
%
% ------
% Author: David Legland
% e-mail: david.legland@grignon.inra.fr
% Created: 2011-06-21,    using Matlab 7.9.0.529 (R2009b)
% Copyright 2011 INRA - Cepia Software Platform.
```

## Data generation

```
% Generate gaussian 3D data
nPoints = 1000;
points = randn(nPoints, 3);

% point clouds parameters
center = [20 30 40];
sizes  = [70 40 10];
orient = [50 30 30];

% transform points to make a gaussian cloud
transfo = composeTransforms3d(...
    createScaling3d(sizes), ...
    eulerAnglesToRotation3d(orient), ...
    createTranslation3d(center));
points = transformPoint3d(points, transfo);

% display data
figure;
drawPoint3d(points, '.');
hold on;
axis equal;
view([30 20]);
```

## Inertia ellipsoid computation and display

```
% Fit a 3D inertia ellipsoid to data
elli = inertiaEllipsoid(points);

% draw the ellipsoid with transparency
drawEllipsoid(elli, 'FaceColor', 'g', 'FaceAlpha', .3);
```

Published with MATLAB® 7.9
