# Supplementary material for: The equatorial position of the metaphase plate ensures symmetric cell divisions
Source: eLife. 2015 Jul 18;4:e05124. doi: 10.7554/eLife.05124 (PMC4536468; doi:10.7554/eLife.05124)
Supplement: Source code 1. — Custom built software in Matlab. DOI: http://dx.doi.org/10.7554/eLife.05124.021 [file elife05124s001.zip › Poles and Kinetochores/External/geom3d/geom3d-demos/html/demoRevolutionSurface.html]

demoRevolutionSurface 

## Contents

- Draw a torus with horizontal axis as revolution axis
- Draw a torus with vertical axis as revolution axis

```
function demoRevolutionSurface(varargin)
```

```
%DEMOREVOLUTIONSURFACE  One-line description here, please.
%   output = demoRevolutionSurface(input)
%
%   Example
%   demoRevolutionSurface
%
%   See also
%
%
% ------
% Author: David Legland
% e-mail: david.legland@nantes.inra.fr
% Created: 2007-04-20
% Copyright 2007 INRA - BIA PV Nantes - MIAJ Jouy-en-Josas.
```

## Draw a torus with horizontal axis as revolution axis

```
circle  = circleToPolygon([10 0 3], 50);
[x y t] = revolutionSurface(circle, linspace(0, 4*pi/3, 50));

figure;
surf(x, y, t);
axis equal;
```

## Draw a torus with vertical axis as revolution axis

```
circle  = circleToPolygon([10 0 3], 50);
revol   = [0 0 0 1];
[x y t] = revolutionSurface(circle, revol, linspace(0, 4*pi/3, 50));

figure;
surf(x, y, t);
axis equal;
```

Published with MATLAB® 7.9
