# Supplementary material for: The equatorial position of the metaphase plate ensures symmetric cell divisions
Source: eLife. 2015 Jul 18;4:e05124. doi: 10.7554/eLife.05124 (PMC4536468; doi:10.7554/eLife.05124)
Supplement: Source code 1. — Custom built software in Matlab. DOI: http://dx.doi.org/10.7554/eLife.05124.021 [file elife05124s001.zip › Poles and Kinetochores/External/geom3d/geom3d-demos/html/drawSoccerBall.html]

drawSoccerBall 

## Contents

- Initialisation
- Draw Faces
- Draw edges

```
function drawSoccerBall
```

```
%DRAWSOCCERBALL  Draw a soccerball using geom3d library
%
%   This demo shows how to use some functions of the geom3d library to
%   compute and display arcs of a soccer ball.
%
%   In particular, it shows how to:
%   * create spheres, circles, circle arcs
%   * create a pre-defined polyhedron
%   * display 3D shapes
%   * compute face centroids
%   * compute intersections of planes, spheres, lines
%   * draw spherical patches
%
%   Usage:
%   drawSoccerBall
%
%
% ------
% Author: David Legland
% e-mail: david.legland@grignon.inra.fr
% Created: 2009-06-22,    using Matlab 7.7.0.471 (R2008b)
% Copyright 2009 INRA - Cepia Software Platform.
```

## Initialisation

```
% origin of 3D basis, which is also the soccerball center
origin = [0 0 0];

% the bounding sphere of the soccer ball, used for computing circles
sphere = [origin 1];

% extract vertices, edges, and faces of soccerball polyhedron
[vertices edges faces] = createSoccerBall;

% prepare figure
figure(1); clf; hold on;
axis equal;

% draw the polyhedron as basis
drawPolyhedron(vertices, faces);
```

## Draw Faces

```
% For each face, draw a spherical surface, composed of several triangular
% patches, with a specific color
for f = 1:length(faces)
    % extract vertices of current face
    faceVertices = faces{f};
    Nvf = length(faceVertices);

    % compute centroid of face vertices
    % (See also function faceCentroids)
    faceCenter = centroid(vertices(faceVertices, :));

    % color of the current face (black for pentagons, white for heaxagons)
    if Nvf == 5
        color = 'k';
    else
        color = 'w';
    end

    % compute and display the spherical face associated with each face
    for i = 1:Nvf
        % current vertices of face edge
        vertex1 = vertices(faceVertices(i), :);
        vertex2 = vertices(faceVertices(mod(i, Nvf)+1), :);

        % draw a portion of the current face
        fillSphericalTriangle(sphere, faceCenter, vertex1, vertex2, ...
            'faceColor', color);
    end
end
```

## Draw edges

```
% For each edge, draw a circle arc on the sphere
for i = 1:size(edges, 1)
    % extremities of current edge
    point1  = vertices(edges(i, 1), :);
    point2  = vertices(edges(i, 2), :);

    % compute plane containing current edge
    plane   = createPlane(origin, point1, point2);

    % intersection of the plane with unit sphere
    circle  = intersectPlaneSphere(plane, sphere);

    % find the position (in degrees) of the 2 vertices on the circle
    angle1  = circle3dPosition(point1, circle);
    angle2  = circle3dPosition(point2, circle);

    % ensure angles are in right direction
    if mod(angle2 - angle1 + 360, 360) > 180
        tmp     = angle1;
        angle1  = angle2;
        angle2  = tmp;
    end

    % compute angle extent of the circle arc
    angleExtent = mod(angle2 - angle1 + 360, 360);

    % create circle arc
    arc = [circle angle1 angleExtent];

    % draw the arc
    drawCircleArc3d(arc, 'linewidth', 2, 'color', 'k');
end

axis off;
axis vis3d;
```

Published with MATLAB® 7.9
