# Supplementary figures and images for: The equatorial position of the metaphase plate ensures symmetric cell divisions
Source: eLife. 2015 Jul 18;4:e05124. doi: 10.7554/eLife.05124 (PMC4536468; doi:10.7554/eLife.05124)

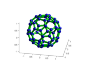

Supplement: Source code 1. — Custom built software in Matlab. DOI: http://dx.doi.org/10.7554/eLife.05124.021 [file elife05124s001.zip › Poles and Kinetochores/External/geom3d/geom3d-demos/html/demoDrawTubularMesh.png]

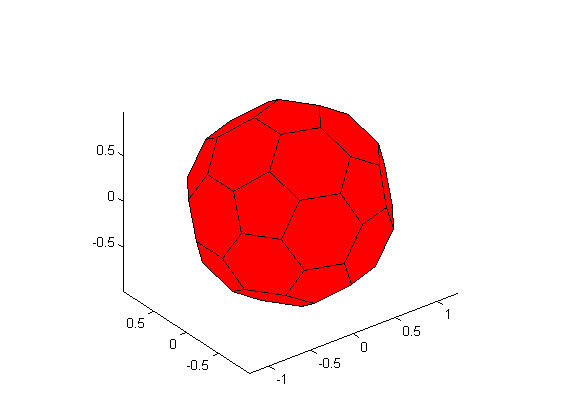

Supplement: Source code 1. — Custom built software in Matlab. DOI: http://dx.doi.org/10.7554/eLife.05124.021 [file elife05124s001.zip › Poles and Kinetochores/External/geom3d/geom3d-demos/html/demoDrawTubularMesh_01.png]

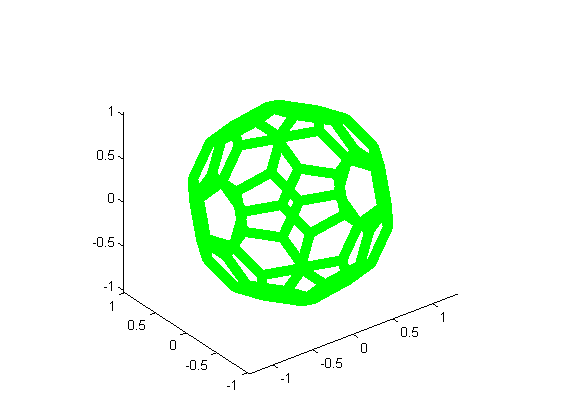

Supplement: Source code 1. — Custom built software in Matlab. DOI: http://dx.doi.org/10.7554/eLife.05124.021 [file elife05124s001.zip › Poles and Kinetochores/External/geom3d/geom3d-demos/html/demoDrawTubularMesh_02.png]

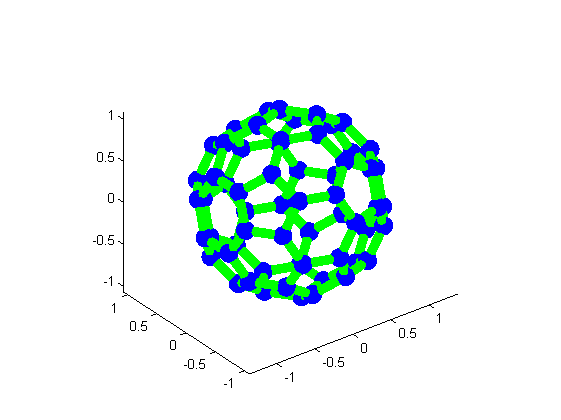

Supplement: Source code 1. — Custom built software in Matlab. DOI: http://dx.doi.org/10.7554/eLife.05124.021 [file elife05124s001.zip › Poles and Kinetochores/External/geom3d/geom3d-demos/html/demoDrawTubularMesh_03.png]

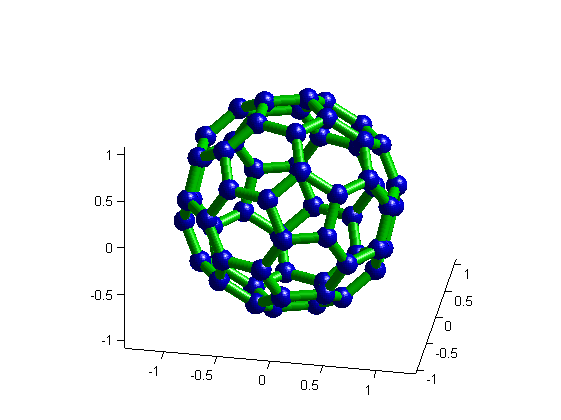

Supplement: Source code 1. — Custom built software in Matlab. DOI: http://dx.doi.org/10.7554/eLife.05124.021 [file elife05124s001.zip › Poles and Kinetochores/External/geom3d/geom3d-demos/html/demoDrawTubularMesh_04.png]

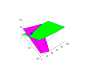

Supplement: Source code 1. — Custom built software in Matlab. DOI: http://dx.doi.org/10.7554/eLife.05124.021 [file elife05124s001.zip › Poles and Kinetochores/External/geom3d/geom3d-demos/html/demoGeom3d.png]

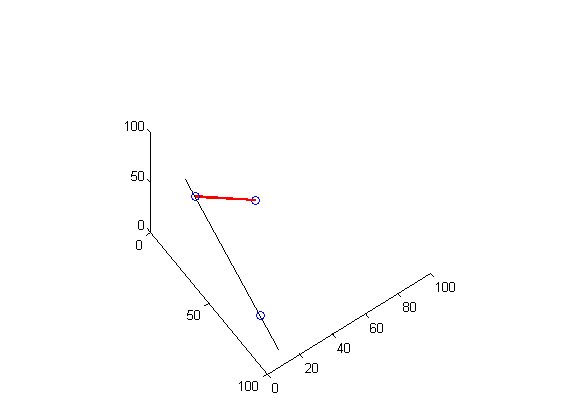

Supplement: Source code 1. — Custom built software in Matlab. DOI: http://dx.doi.org/10.7554/eLife.05124.021 [file elife05124s001.zip › Poles and Kinetochores/External/geom3d/geom3d-demos/html/demoGeom3d_01.png]

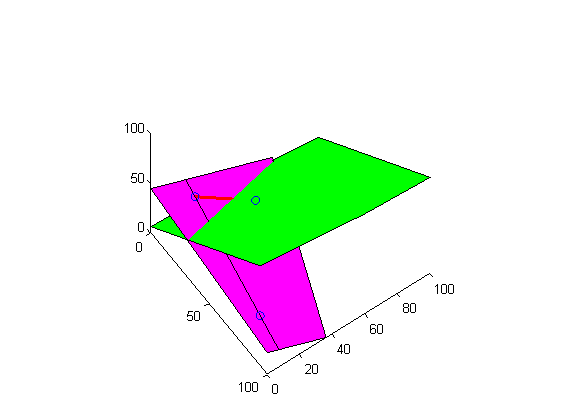

Supplement: Source code 1. — Custom built software in Matlab. DOI: http://dx.doi.org/10.7554/eLife.05124.021 [file elife05124s001.zip › Poles and Kinetochores/External/geom3d/geom3d-demos/html/demoGeom3d_02.png]

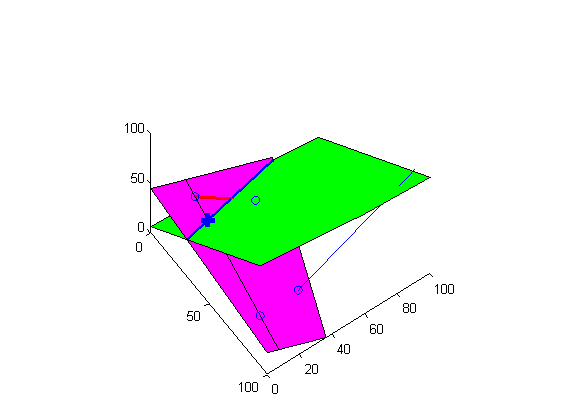

Supplement: Source code 1. — Custom built software in Matlab. DOI: http://dx.doi.org/10.7554/eLife.05124.021 [file elife05124s001.zip › Poles and Kinetochores/External/geom3d/geom3d-demos/html/demoGeom3d_03.png]

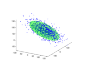

Supplement: Source code 1. — Custom built software in Matlab. DOI: http://dx.doi.org/10.7554/eLife.05124.021 [file elife05124s001.zip › Poles and Kinetochores/External/geom3d/geom3d-demos/html/demoInertiaEllipsoid.png]

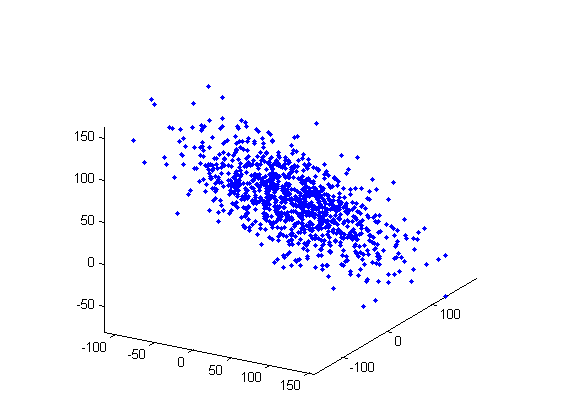

Supplement: Source code 1. — Custom built software in Matlab. DOI: http://dx.doi.org/10.7554/eLife.05124.021 [file elife05124s001.zip › Poles and Kinetochores/External/geom3d/geom3d-demos/html/demoInertiaEllipsoid_01.png]

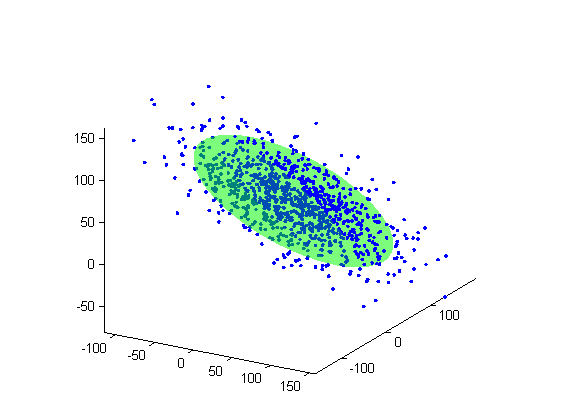

Supplement: Source code 1. — Custom built software in Matlab. DOI: http://dx.doi.org/10.7554/eLife.05124.021 [file elife05124s001.zip › Poles and Kinetochores/External/geom3d/geom3d-demos/html/demoInertiaEllipsoid_02.png]

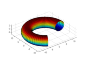

Supplement: Source code 1. — Custom built software in Matlab. DOI: http://dx.doi.org/10.7554/eLife.05124.021 [file elife05124s001.zip › Poles and Kinetochores/External/geom3d/geom3d-demos/html/demoRevolutionSurface.png]

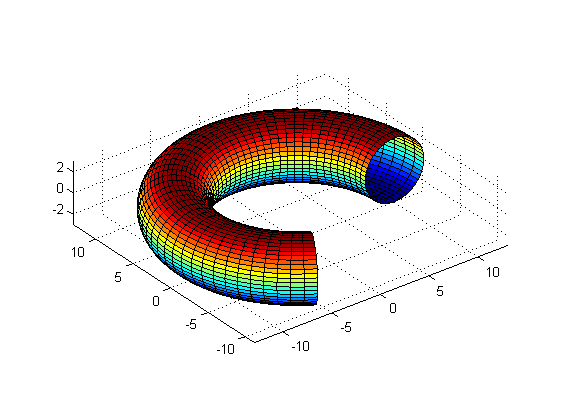

Supplement: Source code 1. — Custom built software in Matlab. DOI: http://dx.doi.org/10.7554/eLife.05124.021 [file elife05124s001.zip › Poles and Kinetochores/External/geom3d/geom3d-demos/html/demoRevolutionSurface_01.png]

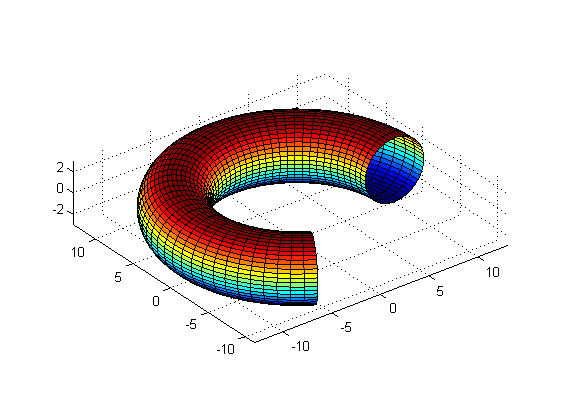

Supplement: Source code 1. — Custom built software in Matlab. DOI: http://dx.doi.org/10.7554/eLife.05124.021 [file elife05124s001.zip › Poles and Kinetochores/External/geom3d/geom3d-demos/html/demoRevolutionSurface_02.png]

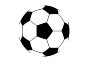

Supplement: Source code 1. — Custom built software in Matlab. DOI: http://dx.doi.org/10.7554/eLife.05124.021 [file elife05124s001.zip › Poles and Kinetochores/External/geom3d/geom3d-demos/html/drawSoccerBall.png]

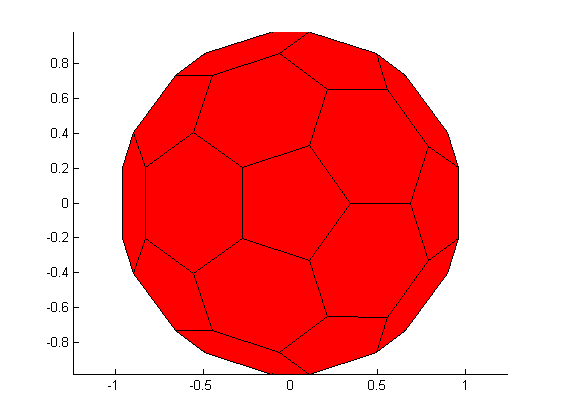

Supplement: Source code 1. — Custom built software in Matlab. DOI: http://dx.doi.org/10.7554/eLife.05124.021 [file elife05124s001.zip › Poles and Kinetochores/External/geom3d/geom3d-demos/html/drawSoccerBall_01.png]

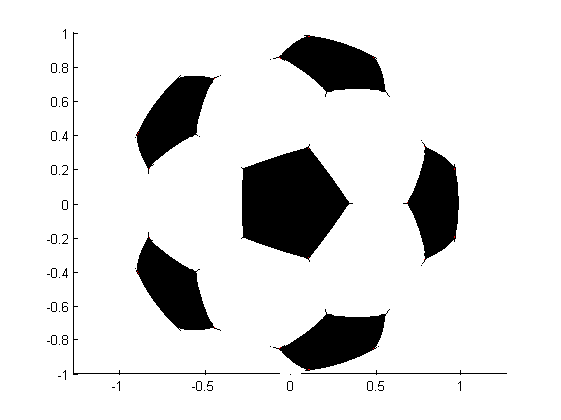

Supplement: Source code 1. — Custom built software in Matlab. DOI: http://dx.doi.org/10.7554/eLife.05124.021 [file elife05124s001.zip › Poles and Kinetochores/External/geom3d/geom3d-demos/html/drawSoccerBall_02.png]

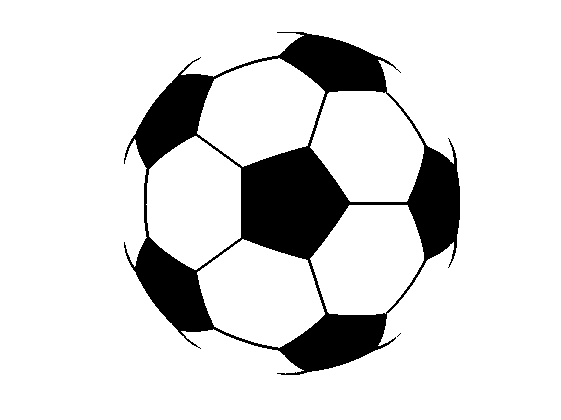

Supplement: Source code 1. — Custom built software in Matlab. DOI: http://dx.doi.org/10.7554/eLife.05124.021 [file elife05124s001.zip › Poles and Kinetochores/External/geom3d/geom3d-demos/html/drawSoccerBall_03.png]
