# Supplementary material for: The equatorial position of the metaphase plate ensures symmetric cell divisions
Source: eLife. 2015 Jul 18;4:e05124. doi: 10.7554/eLife.05124 (PMC4536468; doi:10.7554/eLife.05124)
Supplement: Source code 1. — Custom built software in Matlab. DOI: http://dx.doi.org/10.7554/eLife.05124.021 [file elife05124s001.zip › Poles and Kinetochores/External/geom3d/meshes3d-demos/html/demoPolyhedra.html]

demoPolyhedra 

## Contents

- Cube
- Octahedron
- Cube-Octahedron
- Icosahedron
- Dodecahedron
- Soccer Ball, buckyall, C60...
- Tetrahedron
- Tetrakaidecahedron
- Menger Sponge

```
function demoPolyhedra(varargin)
```

```
%DEMOPOLYHEDRA Draw main polyhedra defined in the matGeom toolbox
%
%   output = demoPolyhedra(input)
%
%   Example
%   demoPolyhedra
%
%   See also
%
%
% ------
% Author: David Legland
% e-mail: david.legland@grignon.inra.fr
% Created: 2010-12-07,    using Matlab 7.9.0.529 (R2009b)
% Copyright 2010 INRA - Cepia Software Platform.
```

## Cube

```
figure(1); clf;
[v f] = createCube;
drawMesh(v, f);
view(3); axis('vis3d'); axis off;
title('Cube');
```

## Octahedron

```
figure(1); clf;
[v f] = createOctahedron;
drawMesh(v, f);
view(3); axis('vis3d'); axis off;
title('Octahedron');
```

## Cube-Octahedron

```
figure(1); clf;
[v f] = createCubeOctahedron;
drawMesh(v, f);
view(3); axis('vis3d'); axis off;
title('Cube-Octahedron');
```

## Icosahedron

```
figure(1); clf;
[v f] = createIcosahedron;
drawMesh(v, f);
view(3); axis('vis3d'); axis off;
title('Icosahedron');
```

## Dodecahedron

```
figure(1); clf;
[v f] = createDodecahedron;
drawMesh(v, f);
view(3); axis('vis3d'); axis off;
title('Dodecahedron');
```

## Soccer Ball, buckyall, C60...

```
figure(1); clf;
[v f] = createSoccerBall;
drawMesh(v, f);
view(3); axis('vis3d'); axis off;
title('Soccer Ball');
```

## Tetrahedron

```
figure(1); clf;
[v f] = createTetrahedron;
drawMesh(v, f);
view(3); axis('vis3d'); axis off;
title('Tetrahedron');
```

## Tetrakaidecahedron

```
figure(1); clf;
[v f] = createTetrakaidecahedron;
drawMesh(v, f);
view(3); axis('vis3d'); axis off;
title('Tetrakaidecahedron');
```

## Menger Sponge

```
figure(1); clf;
[v e f] = createMengerSponge;
drawMesh(v, f);
view(3); axis('vis3d'); axis off;
title('Menger Sponge');
```

Published with MATLAB® 7.9
