# Supplementary material for: The equatorial position of the metaphase plate ensures symmetric cell divisions
Source: eLife. 2015 Jul 18;4:e05124. doi: 10.7554/eLife.05124 (PMC4536468; doi:10.7554/eLife.05124)
Supplement: Source code 1. — Custom built software in Matlab. DOI: http://dx.doi.org/10.7554/eLife.05124.021 [file elife05124s001.zip › Poles and Kinetochores/External/geom3d/meshes3d-demos/html/demoTriangulateFaces.html]

demoTriangulateFaces 

## Contents

- Original polyhedron
- Compute triangulation

```
function demoTriangulateFaces(varargin)
```

```
%TRIANGULATEFACESDEMO Demonstrate how to transform a generic to a trimesh
%   output = demoTriangulateFaces(input)
%
%   Example
%   demoTriangulateFaces
%
%   See also
%
%
% ------
% Author: David Legland
% e-mail: david.legland@nantes.inra.fr
% Created: 2008-10-16,    using Matlab 7.4.0.287 (R2007a)
% Copyright 2008 INRA - BIA PV Nantes - MIAJ Jouy-en-Josas.
```

## Original polyhedron

```
% create a polyhedron with various face vertex numbers
[n e f] = createCubeOctahedron; %#ok<ASGLU>

% display the polyhedron
figure(1); clf;
drawPolyhedron(n, f);

% graphical settings
view(3);
axis equal;
```

## Compute triangulation

```
% Computation of triangulated mesh
tri = triangulateFaces(f);

% display new mesh
figure(2); clf;
patch('vertices', n, 'faces', tri, 'facecolor', 'r');

% graphical settings
view(3);
axis equal;
```

Published with MATLAB® 7.9
