# Supplementary material for: The equatorial position of the metaphase plate ensures symmetric cell divisions
Source: eLife. 2015 Jul 18;4:e05124. doi: 10.7554/eLife.05124 (PMC4536468; doi:10.7554/eLife.05124)
Supplement: Source code 1. — Custom built software in Matlab. DOI: http://dx.doi.org/10.7554/eLife.05124.021 [file elife05124s001.zip › Poles and Kinetochores/External/geom3d/meshes3d-demos/html/demoVoronoiCell.html]

demoVoronoiCell 

## Contents

- Creation of germs
- Display Voronoi polyhedron associated with germ
- Compute and display minimal polyhedron

```
function demoVoronoiCell(varargin)
```

```
%DEMOVORONOICELL Compute and display voronoi polyhedron using geom3d library
%
%   This demo show how to manipulate polyhedra, using matlab's voronoin
%   function and the geom3d library.
%
%   This demo generate some points in a unit cube, computes the 3D voronoi
%   diagram from the germs, and isolate the central cell.
%   This cell is a bounded polyhedron, with polygonal faces. For a better
%   representation of the polyhedron, coplanar faces are merged and a new
%   polyhedron is created and displayed.
%
%   Usage
%   demoVoronoiCell
%
%   See also
%   voronoin, delaunayn, convhulln
%   meshReduce, drawPolyhedron
%
%
% ------
% Author: David Legland
% e-mail: david.legland@grignon.inra.fr
% Created: 2009-06-22,    using Matlab 7.7.0.471 (R2008b)
% Copyright 2009 INRA - Cepia Software Platform.
```

## Creation of germs

```
% germ of the cell of interest, in the center of the unit cube
germ0  = [.5 .5 .5];

% create random germs, and add corners of unit cube to ensure finiteness of
% voronoi pattern
[corners faces] = createCube; %#ok<NASGU>
germs   = [germ0;rand(10, 3);corners];

% dispay the germs
figure(1); clf; hold on;
drawPoint3d(germs);

% graphical settings
view(3);
axis equal;
```

## Display Voronoi polyhedron associated with germ

```
% compute voronoi diagram in 3D using Matlab standard function
[V C]   = voronoin(germs);

% the polyhedron corresponding to first germ is given by vertices in C{1}
indVert = C{1};
vertices = V(indVert, :);

% triangular faces can be obtained from convhulln
K = convhulln(vertices);

% draw the polyhedron
drawPolyhedron(vertices, K);
```

## Compute and display minimal polyhedron

```
% merge some faces to reduce total number of faces.
% new faces are 3D polygons
% K2 is a cell array, each cell containing a row vector with indices of
% 'points' corresponding to vertices of each face.
K2 = meshReduce(vertices, K);

% display the germs
figure(1); clf; hold on;
set(gcf, 'renderer', 'opengl');
drawPoint3d(germs);

% draw the polyhedron
drawPolyhedron(vertices, K2);

% graphical settings
view(3);
axis equal;
```

Published with MATLAB® 7.9
